# Childhood type 1 diabetes: an environment-wide association study across England

**DOI:** 10.1007/s00125-020-05087-7

**Published:** 2020-01-24

**Authors:** Annalisa Sheehan, Anna Freni Sterrantino, Daniela Fecht, Paul Elliott, Susan Hodgson

**Affiliations:** 1 MRC-PHE Centre for Environment and Health, Department of Epidemiology and Biostatistics, Imperial College London, St Mary’s Campus, Norfolk Place, London, W2 1PG UK; 2 School of Population Health and Environmental Sciences, Faculty of Life Sciences and Medicine, King’s College London, London, UK; 3 UK Small Area Health Statistics Unit, School of Public Health, Imperial College London, London, UK; 4 Imperial College NIHR Biomedical Research Centre, Imperial College London, London, UK

**Keywords:** Childhood diabetes, Disease mapping, Environmental exposures, Environment-wide association study, Hospital episode statistics, Routine health data, Type 1 diabetes

## Abstract

**Aims/hypothesis:**

Type 1 diabetes is an autoimmune disease affecting ~400,000 people across the UK. It is likely that environmental factors trigger the disease process in genetically susceptible individuals. We assessed the associations between a wide range of environmental factors and childhood type 1 diabetes incidence in England, using an agnostic, ecological environment-wide association study (EnWAS) approach, to generate hypotheses about environmental triggers.

**Methods:**

We undertook analyses at the local authority district (LAD) level using a national hospital episode statistics-based incident type 1 diabetes dataset comprising 13,948 individuals with diabetes aged 0–9 years over the period April 2000 to March 2011. We compiled LAD level estimates for a range of potential demographic and environmental risk factors including meteorological, land use and environmental pollution variables. The associations between type 1 diabetes incidence and risk factors were assessed via Poisson regression, disease mapping and ecological regression.

**Results:**

Case counts by LAD varied from 1 to 236 (median 33, interquartile range 24–46). Overall type 1 diabetes incidence was 21.2 (95% CI 20.9, 21.6) per 100,000 individuals. The EnWAS and disease mapping indicated that 15 out of 53 demographic and environmental risk factors were significantly associated with diabetes incidence, after adjusting for multiple testing. These included air pollutants (particulate matter, nitrogen dioxide, nitrogen oxides, carbon monoxide; all inversely associated), as well as lead in soil, radon, outdoor light at night, overcrowding, population density and ethnicity. Disease mapping revealed spatial heterogeneity in type 1 diabetes risk. The ecological regression found an association between type 1 diabetes and the living environment domain of the Index of Multiple Deprivation (RR 0.995; 95% credible interval [CrI] 0.991, 0.998) and radon potential class (RR 1.044; 95% CrI 1.015, 1.074).

**Conclusions/interpretation:**

Our analysis identifies a range of demographic and environmental factors associated with type 1 diabetes in children in England.

**Electronic supplementary material:**

The online version of this article (10.1007/s00125-020-05087-7) contains peer-reviewed but unedited supplementary material, which is available to authorised users.

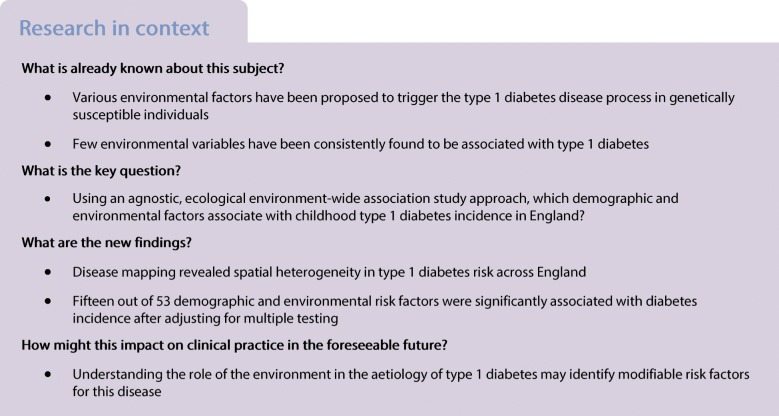



## Introduction

Type 1 diabetes is an autoimmune disease resulting in the destruction of pancreatic insulin-secreting beta cells [1]. The majority of people with type 1 diabetes require lifelong insulin replacement therapy and have reduced life expectancy and quality of life; their treatment places a substantial economic burden on health services, with direct UK health costs estimated at £1bn in 2010–2011 [2].

Genetic predisposition plays a role in the development of type 1 diabetes. Although more than 40 risk loci are associated with type 1 diabetes [3], most individuals who possess type 1 diabetes risk genes do not, however, develop diabetes [3–5], suggesting additional factors are needed to trigger and drive the disease process.

Various environmental triggers of type 1 diabetes have been proposed [3–5]. Viral infections have long been associated with type 1 diabetes [6]. Several studies have reported finding enteroviruses, anti-enterovirus antibodies or enterovirus RNA more frequently in diabetic individuals than in healthy individuals [7]. A viral aetiology is also supported by the observed spatial and/or temporal clustering of type 1 diabetes [8], seasonal variation in onset [9], and various social and demographic factors that relate to population mixing (and potential for infection), e.g. urban/rural status, remoteness, population density, overcrowding and socioeconomic status/social class [10, 11]. Other, non-infectious environmental variables have also been implicated, including levels of nitrates in drinking water [12] and meteorological factors such as sunshine duration and temperature [13].

We aimed to identify environmental correlates of childhood type 1 diabetes in England, using an agnostic, ecological environment-wide association study (EnWAS) approach to generate hypotheses about potential environmental triggers of type 1 diabetes for testing in further studies.

## Methods

### Health data

We obtained type 1 diabetes data from National Health Service (NHS) Digital hospital episode statistics (HES) records (https://digital.nhs.uk/data-and-information/data-tools-and-services/data-services/hospital-episode-statistics) held by the UK Small Area Health Statistics Unit. We identified all inpatient admissions for children aged 0–9 years with a primary diagnosis of type 1 or type unknown diabetes (ICD-9 codes: 250X1, 250X3, 250X, 250X9 [www.icd9data.com/2007/Volume1]; ICD-10 codes: E10X, E12X, E13X, E14X [http://apps.who.int/classifications/icd10/browse/2016/en]) admitted between 1 January 1992 and 31 March 2011, to create a dataset of incident diabetes during the period 1 April 2000 to 31 March 2011 based on first admission for each unique patient [14]. Readmissions were excluded on the basis of the unique patient identifier (HES-ID) and for those with missing HES-ID (predominantly affecting admissions prior to 1997), from unique combinations of date of birth, sex and pseudonymised postcode. We summed observed counts to the 2001 local authority district (LAD) level (*n* = 354 LADs in England, mean population ~140,000) and calculated age- and sex-adjusted expected counts using the England-wide dataset as the reference.

### Environmental data

We generated LAD level estimates of exposure to a range of environmental factors, as summarised below and in electronic supplementary material (ESM) Table 1. We used the Office for National Statistics 2001 postcode population headcounts for population weighting, and analyses were conducted in ArcGIS (Environmental Systems Research Institute, Redlands, CA, USA, 2017).

#### Meteorological conditions

We calculated population-weighted LAD level daily mean sunshine duration for 1980–2005 at a 1 × 1 km resolution using data supplied by the British Atmospheric Data Centre [15]. We calculated population-weighted LAD level annual minimum and maximum temperatures using the Met Office UKCP09 dataset containing monthly mean daily minimum and maximum temperatures for 1981–2010 on a 5 km grid point scale. We used population weighting of monthly mean pre-vitamin D action spectrum ultraviolet for postcode districts, from July 2002 to June 2003, to calculate LAD level annual mean ultraviolet radiation [16].

#### Built environment

We summed the percentage of the total area of each LAD comprising green space, blue space, built environment and intense agriculture using the 25× 25 m resolution Centre for Ecology and Hydrology land cover map 2000 [17]. Using Ordnance Survey greenspace data [18], we calculated the percentage of each LAD’s population that met Natural England’s accessible natural greenspace standard (ANGSt) criteria of: (1) at least 2 hectares of greenspace, no more than 300 m (5 min walk) from home; (2) at least one accessible 20 hectare site within 2 km of home; (3) one accessible 100 hectare site within 5 km of home; (4) one accessible 500 hectare site within 10 km of home [19]. We calculated the proportion of population in each LAD, classified as rural or urban using the Department for Environment, Food and Rural Affairs (Defra) rural/urban classification for 2001 [20].

#### Environmental pollutants

We calculated the population-weighted LAD level mean radon potential class using 1 × 1 km resolution data from the Public Health England–British Geological Survey 2007 Indicative Atlas of Radon in the United Kingdom [21]. We modelled population-weighted LAD level annual mean estimates of background particulate matter with an aerodynamic diameter <10 μm (PM_10_), nitrogen dioxide and nitrogen oxides (2001); particulate matter with an aerodynamic diameter <2.5 μm (PM_2.5_), carbon monoxide and sulphur dioxide (2002); and ozone and benzene (2003) using 1 × 1 km background pollution data from Defra [22]. We also derived population-weighted annual mean nitrogen dioxide and PM_10_ (2007) from an EU-wide land use regression model (100 × 100 m) [23] to check for consistency across different models. We calculated population-weighted LAD level lead, cadmium and arsenic in soil via empirical Bayesian kriging using the British Geological Survey’s Geochemical Baseline Survey of the Environment [24] and National Soil Inventory soils summary information [25]. We calculated population-weighted LAD level estimates of nitrates in drinking water (2000–2010) using data from 14 water companies (Anglian Water, Bristol Water, South Staffs Water, Northumbrian Water, Portsmouth Water, Severn Trent Water, SES Water, Southern Water, South East Water, South West Water, Thames Water, United Utilities, Dŵr Cymru Welsh Water and Wessex Water). We calculated population-weighted LAD level agricultural farmland use of six different groups of pesticides (fungicides, herbicides and desiccants, growth regulators, insecticides/nemacides/acaracides, molluscicides and repellents, and other) and total pesticide use using data from the Integrated Assessment of Health Risks of Environmental Stressors in Europe project [26], derived from Defra’s June 2000 Agricultural Returns census and the Pesticides Usage Survey carried out by the Food and Environment Research Agency (now Fera Science). We calculated population-weighted LAD level estimates of light at night, in deciles, using data produced as part of the Mapping Night-time Light Emissions project [27] available at 200 × 200 m resolution.

#### Demographic characteristics

We calculated LAD level population density (people per km^2^) using the Office for National Statistics LAD level mid-year population estimates for 2000 and 2001 [28]. We calculated per cent overcrowded households per LAD using 2001 census data [29]. We assigned population-weighted LAD level 2004 Index of Multiple Deprivation (IMD) and domains of IMD (income deprivation; employment deprivation; health deprivation and disability; education, skills and training deprivation; barriers to housing and services; crime; the living environment) using data from the Department for Communities and Local Government [30] and the method proposed by McLennan et al. [31]. We aggregated LAD level weekly tobacco expenditure per person ≥16 years of age, obtained from CACI (London, UK, www.caci.co.uk) 2014 census output area data. We assigned LAD level percentages of white (white British, white Irish, white other), black (black Caribbean, black African, black) and Asian (Indian, Pakistani and Bangladeshi) ethnicity using 2001 census data.

### Statistical methods

To assess the association between type 1 diabetes and the 53 environmental factors, we proceeded in stages. In the first stage, using a frequentist EnWAS approach, we fitted for each environmental variable a LAD level univariable Poisson regression with the type 1 diabetes case count as the dependent variable and the age- and sex-adjusted expected count as the offset. To account for multiple testing, we applied a Bonferroni correction control procedure. The EnWAS results are presented in a Manhattan plot, which shows the –log_10_(*p* value) × sign of association, i.e. the statistical significance and direction, but not magnitude, of the unadjusted association between each variable and type 1 diabetes incidence.

In the second stage, we fitted, for each environmental variable, the same univariable Poisson regression models but in a Bayesian framework, to include the spatial dependency between neighbouring LADs (‘disease mapping’), and reported the 95% credible intervals (CrIs) [32]. We mapped the spatial residual RR to identify areas of high and low risk, jointly with posterior probability maps as a measure of uncertainty (i.e. to identify areas with an 80% probability of risk being higher or lower than the national mean).

In the third stage, we fitted a multivariable Poisson ecological regression, which included those environmental risk factors that presented an adjusted *p* value <0.05 in the EnWAS and a relevant CrI (i.e. RR >1 or <1) from the disease mapping. As many of the risk factors were correlated, we created a heat map to identify key variables from each correlated ‘group’ of variables (many of the air pollutants, for instance, were correlated such that they could not be included together in the same model) for inclusion in the ecological regression. Disease mapping and ecological regression accounted for the spatial random effect and were fitted using R-INLA (www.r-inla.org [33]), with a variation of the Besag–York–Mollie model [34] that allows estimation of a mixing variable to explain how much variability is due to spatial component or over-dispersion in the data [35].

### Ethics approval

The study used Small Area Health Statistics Unit data, obtained from NHS Digital and the Office for National Statistics. The study was covered by national research ethics approval from the London–South East Research Ethics Committee (reference 17/LO/0846). Data access was covered by the Health Research Authority Confidentiality Advisory Group under section 251 of the National Health Service Act 2006 and the Health Service (Control of Patient Information) Regulations 2002 – HRA CAG reference: 14/CAG/1039.

## Results

The HES-derived dataset included 13,948 incident type 1 diabetes cases aged 0–9 years and first admitted to hospital with a type 1 diabetes diagnosis over the period April 2000 to March 2011. Case counts by LAD varied from 1 to 236 (median 33, interquartile range 24–46). The overall incidence was 21.2 (95% CI 20.9, 21.6) per 100,000; age- and sex-standardised incidence rates ranged from 4.45 to 80.55 per 100,000.

**Fig. 1:**
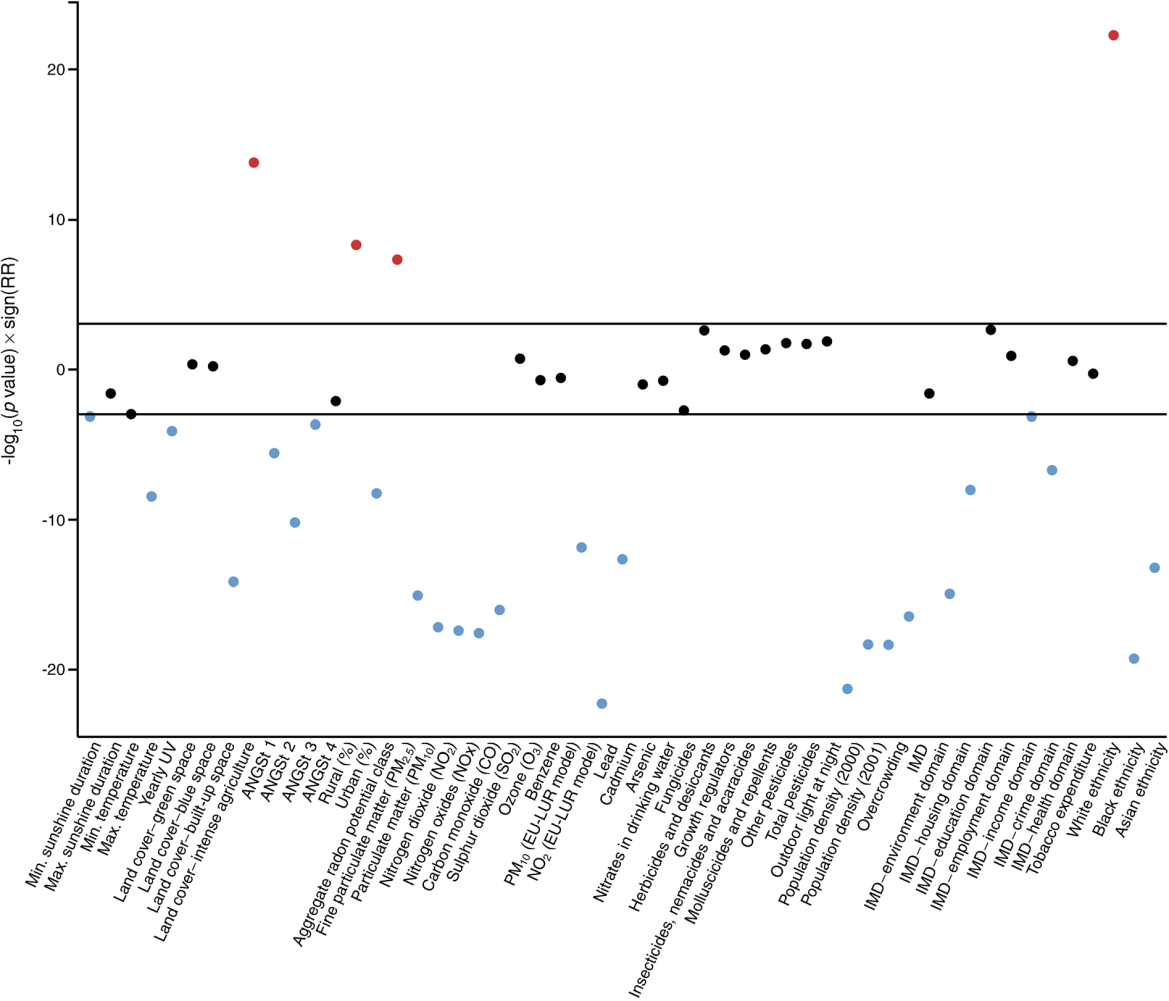
Manhattan plot of associations between the 53 demographic and environmental variables and type 1 diabetes, where variables with log_10_-transformed *p* values above and below the black lines (Bonferroni correction) are statistically associated with type 1 diabetes (red, positively; blue, negatively). ANGSt criteria are defined in the Methods section; ‘sign’ indicates the sign of association, such that the plot shows the statistical significance and direction, but not magnitude, of the unadjusted association between each variable and type 1 diabetes incidence; EU-LUR, European land use regression air pollution model; UV, ultraviolet

**Fig. 2:**
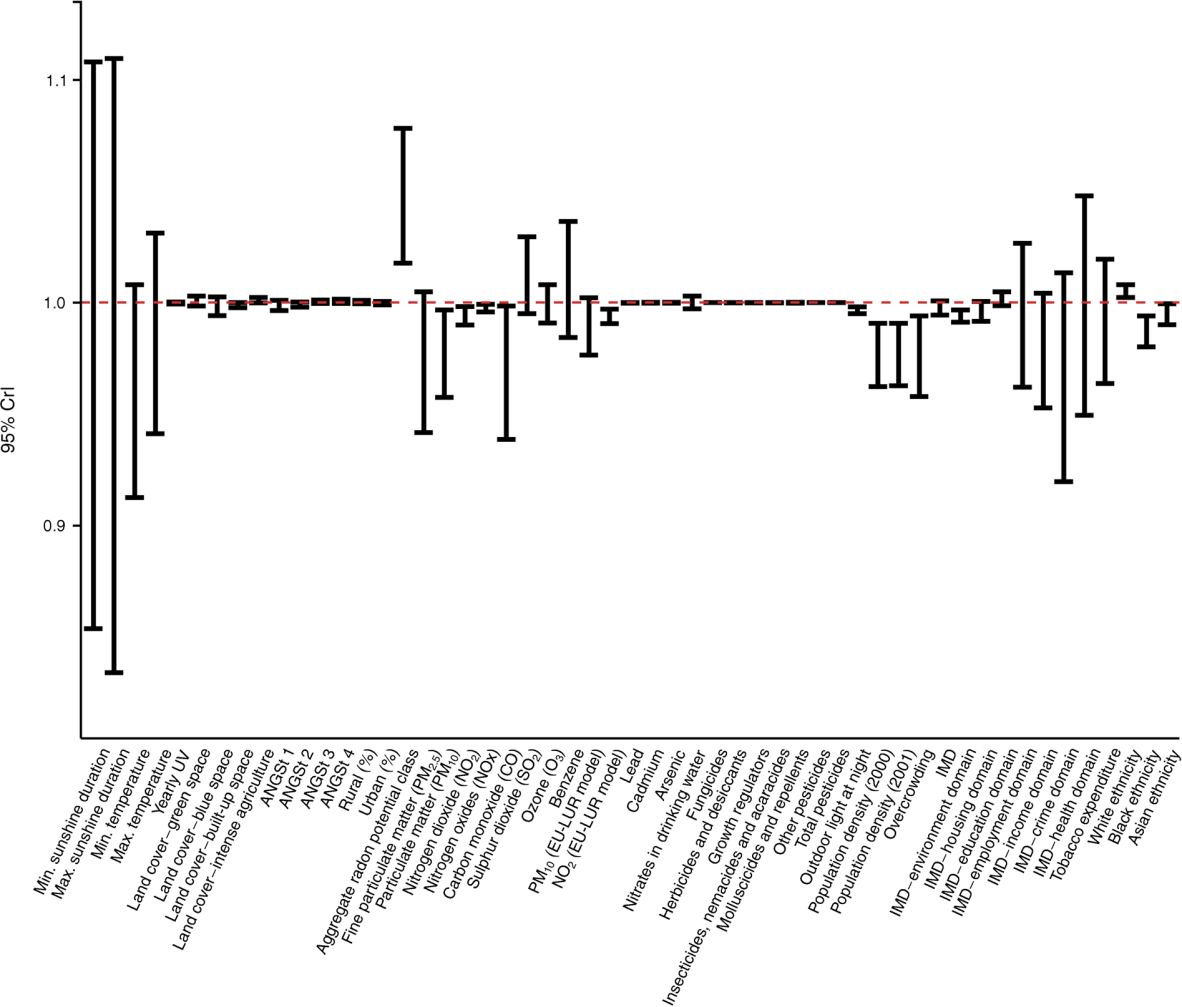
CrIs for RRs for each of the environmental variables from the Bayesian ecological regression, where those with 95% CrIs >1 or <1 (red dashed line) are statistically associated with type 1 diabetes. ANGSt criteria are defined in the Methods section; EU-LUR, European land use regression air pollution model; UV, ultraviolet

The Manhattan plot (Fig. 1) shows that 30 of the 53 environmental variables were significantly associated with type 1 diabetes after applying the Bonferroni correction for multiple testing (*p* < 0.0009); all but four showed negative associations. A quantile–quantile plot of the –log_10_(*p* values) (ESM Fig. 1) shows an S-shaped plot suggesting under-dispersed data, and deviation from expectation under the null. Figure 2 plots the CrIs from the Bayesian Poisson regression (disease mapping), which additionally accounts for the spatial dependency between areas. Fifteen of the 30 variables that were statistically significantly associated with type 1 diabetes incidence in the frequentist EnWAS were also found to be significantly associated with type 1 diabetes after accounting for spatial dependency in the data (Table 1).

**Table 1:** Direction, magnitude and significance of the association with type 1 diabetes incidence for each environmental variable for the frequentist ecological EnWAS and Bayesian disease-mapping approach

Variable	EnWAS			Disease mapping	
	Mean	95% CI	Adjusted *p* value^a^	Mean	95% CrI
Minimum sunshine duration	0.920	0.877, 0.966	0.001	0.973	0.854, 1.108
Maximum sunshine duration	0.938	0.888, 0.991	0.023	0.963	0.835, 1.109
Minimum temperature	0.956	0.931, 0.982	0.001	0.959	0.913, 1.008
Maximum temperature	0.959	0.946, 0.972	<0.001	0.985	0.941, 1.031
Yearly ultraviolet radiation	1.000	1.000, 1.000	<0.001	1.000	0.999, 1.000
Land cover					
Green space	1.000	0.999, 1.002	0.487	1.001	0.998, 1.003
Blue space	1.001	0.998, 1.003	0.636	0.998	0.994, 1.003
Built-up space	0.997	0.997, 0.998	<0.001	0.999	0.998, 1.000
Intense agriculture	1.003	1.002, 1.003	<0.001	1.001	1.000, 1.002
ANGSt 1^b^	0.996	0.995, 0.998	<0.001	0.999	0.996, 1.001
ANGSt 2^b^	0.998	0.997, 0.998	<0.001	0.999	0.998, 1.000
ANGSt 3^b^	0.999	0.999, 1.000	<0.001	1.000	1.000, 1.001
ANGSt 4^b^	0.999	0.999, 1.000	0.007	1.001	1.000, 1.002
Rural status, %	1.001	1.001, 1.002	<0.001	1.000	1.000, 1.001
Urban status, %	0.999	0.998, 0.999	<0.001	1.000	0.999, 1.000
Aggregate radon potential class^c^	1.049	1.031, 1.067	<0.001	1.047	1.018, 1.078
Fine particulate matter (PM_2.5_)	0.939	0.925, 0.954	<0.001	0.972	0.942, 1.005
Particulate matter (PM_10_)^c^	0.958	0.949, 0.968	<0.001	0.977	0.958, 0.997
Nitrogen dioxide^c^	0.990	0.988, 0.993	<0.001	0.994	0.990, 0.998
Nitrogen oxides^c^	0.996	0.995, 0.997	<0.001	0.997	0.996, 0.999
Carbon monoxide^c^	0.930	0.914, 0.946	<0.001	0.968	0.939, 0.999
Sulphur dioxide	1.006	0.997, 1.015	0.198	1.012	0.995, 1.030
Ozone	0.998	0.995, 1.001	0.179	0.999	0.991, 1.008
Benzene	0.989	0.969, 1.008	0.261	1.010	0.984, 1.036
PM_10_ (EU-LUR model)	0.976	0.970, 0.983	<0.001	0.989	0.977, 1.002
Nitrogen dioxide (EU-LUR model)^c^	0.992	0.990, 0.993	<0.001	0.994	0.991, 0.997
Lead^c^	0.999	0.999, 0.999	<0.001	0.999	0.999, 0.999
Cadmium	1.000	1.000, 1.000	0.096	1.000	1.000, 1.000
Arsenic	1.000	1.000, 1.000	0.165	1.000	1.000, 1.000
Nitrates in drinking water	0.997	0.996, 0.999	0.002	1.000	0.997, 1.003
Fungicides	1.000	1.000, 1.000	0.003	1.000	1.000, 1.000
Herbicides and desiccants	1.000	1.000, 1.000	0.055	1.000	1.000, 1.000
Growth regulators	1.000	1.000, 1.000	0.110	1.000	1.000, 1.000
Insecticides, nemacides and acaracides	1.000	1.000, 1.000	0.048	1.000	1.000, 1.000
Molluscicides and repellents	1.000	1.000, 1.000	0.018	1.000	1.000, 1.000
Other pesticides	1.000	1.000, 1.000	0.020	1.000	1.000, 1.000
Total pesticides	1.000	1.000, 1.000	0.014	1.000	1.000, 1.000
Outdoor light at night^c^	0.996	0.995, 0.996	<0.001	0.997	0.995, 0.998
Population density (2000)^c^	0.964	0.957, 0.972	<0.001	0.976	0.963, 0.991
Population density (2001)^c^	0.965	0.957, 0.972	<0.001	0.977	0.963, 0.991
Overcrowding^c^	0.959	0.949, 0.968	<0.001	0.976	0.958, 0.994
IMD	0.998	0.996, 1.000	0.024	0.998	0.994, 1.001
Living environment domain^c^	0.994	0.992, 0.995	<0.001	0.994	0.991, 0.997
Housing domain	0.993	0.991, 0.995	<0.001	0.996	0.992, 1.001
Education domain	1.003	1.001, 1.005	0.002	1.002	0.999, 1.005
Employment domain	1.012	0.996, 1.028	0.130	0.994	0.962, 1.027
Income domain	0.975	0.961, 0.989	0.001	0.978	0.953, 1.004
Crime domain	0.924	0.896, 0.952	<0.001	0.965	0.920, 1.013
Health domain	1.013	0.989, 1.038	0.284	0.997	0.950, 1.048
Tobacco expenditure	0.995	0.982, 1.009	0.503	0.991	0.964, 1.020
White ethnicity^c^	1.008	1.006, 1.009	<0.001	1.005	1.002, 1.008
Black ethnicity^c^	0.982	0.978, 0.986	<0.001	0.987	0.980, 0.994
Asian ethnicity^c^	0.989	0.986, 0.992	<0.001	0.995	0.990, 1.000

^a^*p* < 0.0009 to be significant after Bonferroni correction for multiple testing

^b^ANGSt criteria are defined in the Methods section

^c^Variable significantly associated with type 1 diabetes across both approaches

EU-LUR, European land use regression air pollution model

**Fig. 3:**
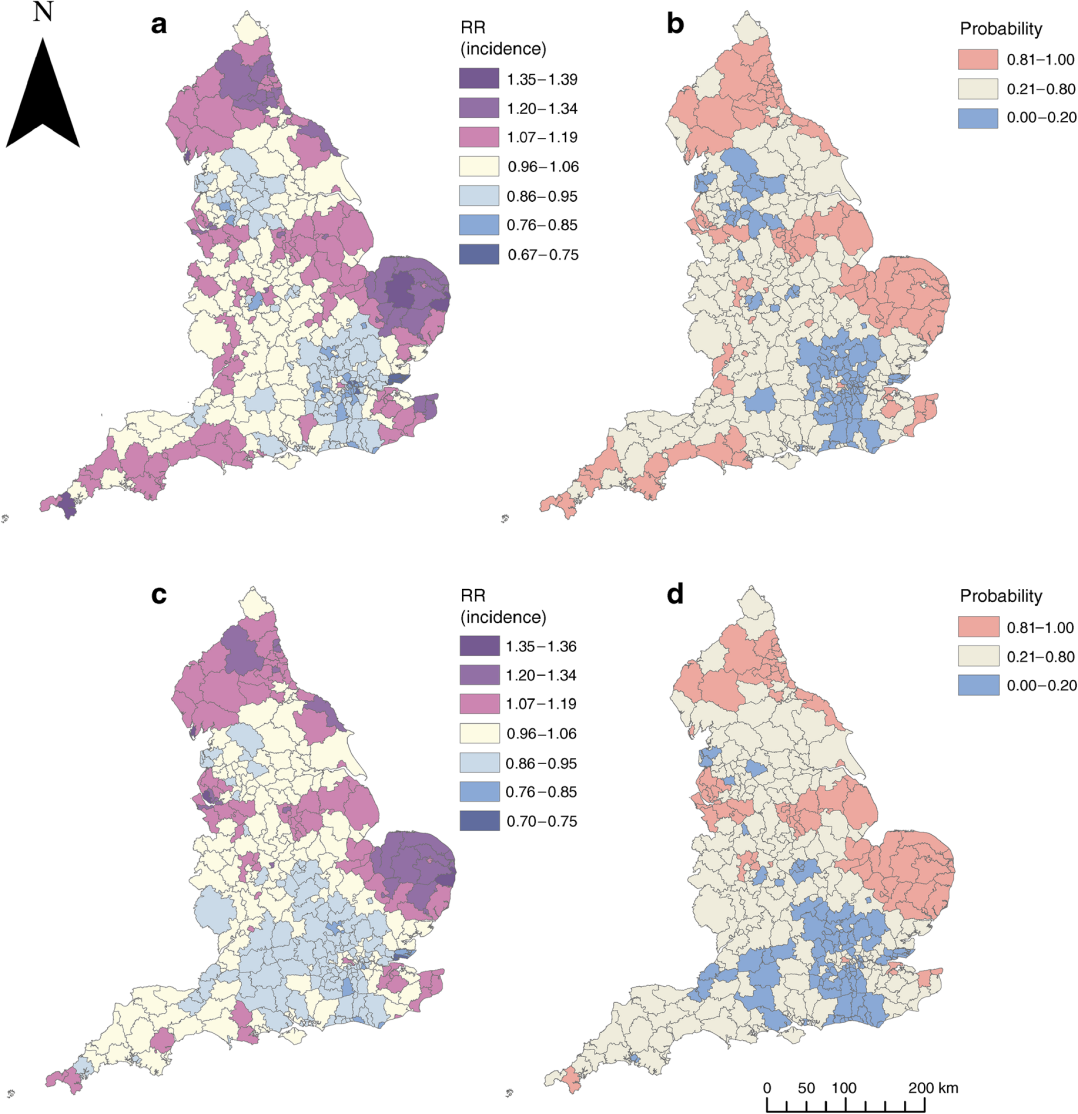
Type 1 diabetes incidence in children aged 0–9 years, adjusted for age and sex, 2000–2011, at LAD level in England. (**a**) Smoothed RRs and (**b**) posterior probabilities, from disease mapping in R-INLA. (**c**) Smoothed RRs and (**d**) posterior probabilities, from the ecological regression model including nitrogen dioxide, lead in soil, aggregate radon potential, black ethnicity, overcrowding and IMD living environment domain

The RR of type 1 diabetes by LAD varied from 0.68 to 1.39 across England, with higher RRs (and those with an 80% probability of risks being higher than the England mean) appearing to be in coastal and more rural areas, notably Norfolk, the North East and Cornwall (Fig. 3a, b). However, rural/urban status was only associated with type 1 diabetes incidence in the EnWAS; this finding was not replicated in the disease-mapping analysis. A large proportion of the variability in type 1 diabetes (78%) was explained by the spatial structured component. The spatial pattern was consistent over time: LAD level observed counts for 2000–2005 vs 2006–2011 were highly correlated (Pearson correlation 0.885).

**Fig. 4:**
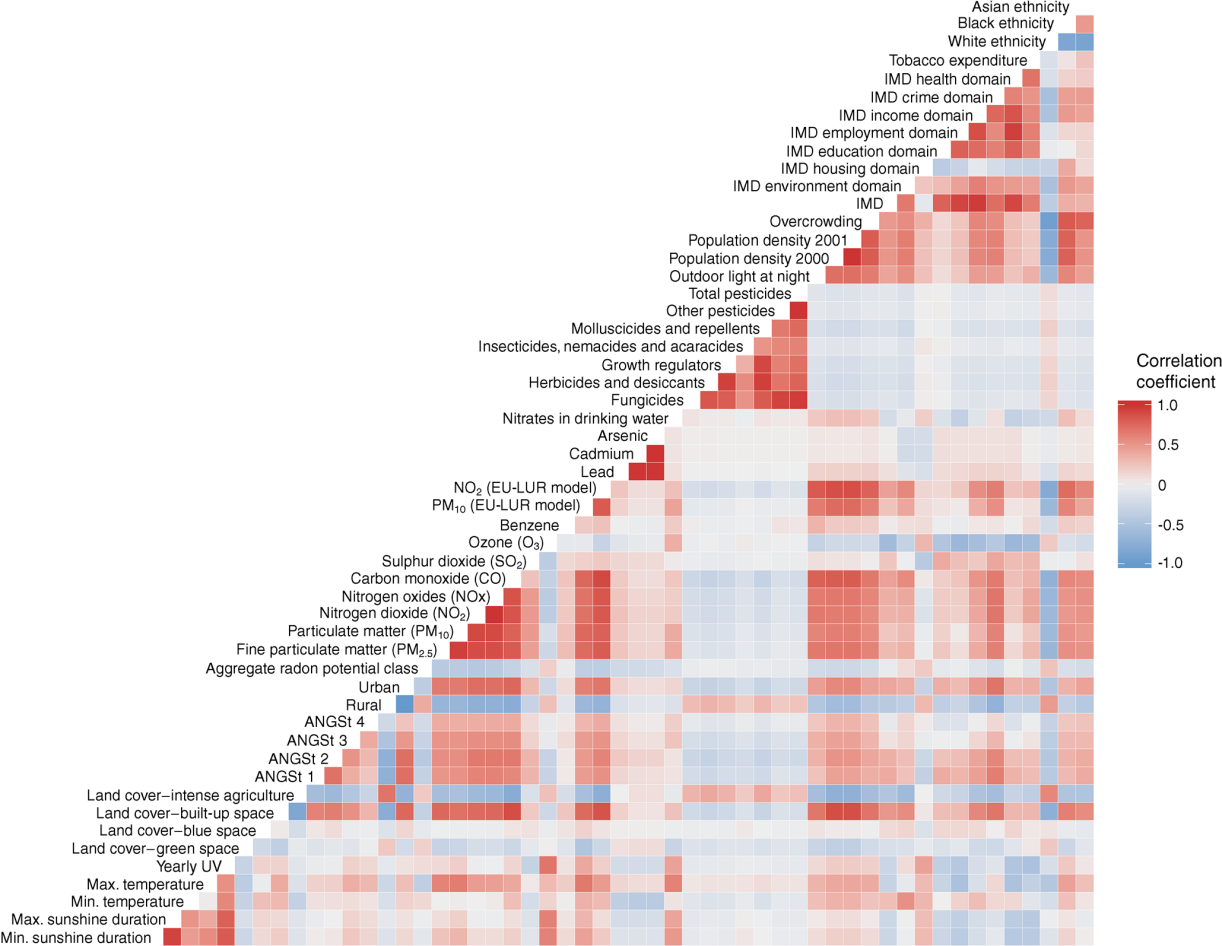
Spearman’s rank correlation heat map for the demographic and environmental variables. ANGSt criteria are defined in the Methods section; EU-LUR, European land use regression air pollution model; UV, ultraviolet

The heat map (Fig. 4) shows the correlations between the 53 variables investigated. As the air pollutants, and many of the demographic variables, were highly intercorrelated, they could not be included in the same model. We developed an ecological regression selecting non- (or less) correlated variables from among the 15 variables significantly associated with type 1 diabetes in both the EnWAS and disease mapping (Table 2). We included nitrogen dioxide (as a marker of air pollution), lead in soil, radon potential class, ethnicity, overcrowding and IMD living environment domain in the ecological regression and found an association with the living environment (RR 0.995; 95% CrI 0.991, 0.998) and radon potential class (RR 1.044; 95% CrI 1.015, 1.074), with 85% of the variability in type 1 diabetes risk explained by the spatial structured component. With adjustment for these variables we identified fewer LADs with an 80% probability of having a higher (74/354 vs 87/354) or lower (79/354 vs 88/354) risk compared with England as a whole (Fig. 3d, b). Models with PM_10_ instead of nitrogen dioxide, or with Asian ethnicity instead of black ethnicity, and removal of lead in soil (due to correlations with nitrogen dioxide/PM_10_/ethnicity) did not materially alter the output.

**Table 2:** Ecological regression for childhood type 1 diabetes risk, adjusted for age and sex

Variable	RR	95% CrI
Nitrogen dioxide	1.000	0.995, 1.005
Lead in soil	0.999	0.999, 1.000
Aggregate radon potential	1.044	1.015, 1.074
Black ethnicity	0.991	0.981, 1.001
Overcrowding	1.015	0.988, 1.044
IMD living environment domain	0.995	0.991, 0.998

## Discussion

In our England-wide small area study, using data from nearly 14,000 childhood type 1 diabetes cases, we found marked spatial heterogeneity in type 1 diabetes risk and identified several environmental variables associated with type 1 diabetes incidence which might warrant further investigation. Pollution and demographic variables associated with urbanicity, including air pollution, light at night and lead in soil, along with population density, overcrowding and minority ethnic populations, were all negatively associated with type 1 diabetes incidence in the age- and sex-adjusted analyses, although these variables did not remain significant in a multivariable ecological regression. The significant negative association we observed between type 1 diabetes and the IMD living environment domain and the positive association with radon potential class did remain significant in the multivariable ecological regression. However, this model did not explain the observed spatial structure in area level incidence, suggesting other factors also play a role.

Ethnic differences in incidence have been reported previously, including in England, where Harron et al. found the incidence of type 1 diabetes in non-South Asians to be significantly higher than in South Asians in Yorkshire [36]. In Harron et al.’s individual level study the ethnicity of the type 1 diabetes cases was known, whereas in our area level study the observed increase in type 1 diabetes incidence was in areas with a higher proportion of white ethnic population, not necessarily in people of white ethnicity.

In our study, living environment deprivation was the only domain of IMD significantly (negatively) associated with type 1 diabetes, after accounting for the spatial dependency in the data. This IMD domain describes deprivation in the ‘indoors’ living environment (specifically social and private housing in poor condition and houses without central heating) and ‘outdoors’ living environment (comprising an overall air quality score and road traffic accidents involving injury to pedestrians and cyclists) [37]. We did not find any previous studies looking at type 1 diabetes incidence and housing conditions, but previous studies have looked at type 1 diabetes with respect to composite measures of deprivation which might correlate with components of the IMD indoor living environment domain. For example, Staines et al. and Crow et al. reported a reduced risk of type 1 diabetes in children with decreasing levels of deprivation in West Yorkshire, North Yorkshire and Humberside [11] and northern England [10].

With respect to overcrowding, previous studies in Northern Ireland [38] and West/North Yorkshire and Humberside [11] reported negative associations between type 1 diabetes risk and overcrowding. Our finding of a negative association between overcrowding and type 1 diabetes is in keeping with these earlier studies, although overcrowding did not remain significant in our multivariable ecological regression.

Previous studies have reported inconsistent findings on the association between type 1 diabetes and air pollution. In Southern California, pre-diagnosis ozone and PM_10_ exposure was significantly higher in people with type 1 diabetes diagnosed before 5 years of age, although nitrogen dioxide, sulphur dioxide and sulphate exposures were significantly lower in the later onset group [39]; a significantly higher OR was found for cumulative exposure to ozone and sulphate [40]. In Bavaria, Germany, exposure to PM_10_ and nitrogen dioxide was reported to accelerate the manifestation of type 1 diabetes [41]. Our area level observations of a negative association between type 1 diabetes and air pollution do not corroborate these findings.

We found a small but significant negative association between type 1 diabetes and lead in soil in our EnWAS and Bayesian analyses, although this association did not remain significant in the multivariable ecological regression. A study in Sardinia reported a significant negative correlation between type 1 diabetes incidence rates and lead in stream sediments [42].

We found a negative association between type 1 diabetes and both light at night and population density in our EnWAS and Bayesian analyses, although these variables are highly correlated [43]. A negative association between type 1 diabetes and population density has been observed elsewhere [11, 38], although others have reported a positive association [44, 45]. Researchers have theorised that population density and overcrowding might associate with type 1 diabetes via a viral aetiology and/or hygiene hypothesis [5–7], although population density is also closely associated with other urban phenomena, including air pollution, ethnicity and land contamination (e.g. lead in soil), as discussed above.

We found a consistent, significant positive association between radon potential class and type 1 diabetes risk across all our analyses. In the UK, higher radon potential tends to be found in rural areas, especially in South West England and Wales [46]. Our aggregate measure of radon potential was calculated from Public Health England–British Geological Survey radon data, where each 1 km grid square is classed according to the percentage of homes (0–1%, 1–3%, 3–5%, 5–10%, 10–30% or >30%) within each grid square predicted to be above the action level of 200 Bq/m^3^, which does not convert well to an aggregate, population-weighted ecological measure of radon exposure. We could find no other studies specifically linking radon exposure to type 1 diabetes to support our finding.

In our ecological analyses we found little evidence of an association between type 1 diabetes and the meteorological variables sunshine hours, temperature or ultraviolet B exposure. Previous studies reporting such associations have often taken a continental or global approach [47], where variation in these variables will be considerably greater than those observed across England. We found no evidence to support an association between type 1 diabetes and nitrates in drinking water. The mean LAD level nitrate concentration we calculated across England was 17 mg/l (range 0.02–39 mg/l), which is below the WHO drinking water guideline value of 50 mg/l. Nonetheless, 194 of the 319 LADs where nitrate levels could be assessed had mean nitrate levels above the 14.85 mg/l upper tertile of nitrate levels at which a significantly increased risk was observed in Yorkshire [12]. Our inability to observe an association between nitrates in drinking water and type 1 diabetes is unlikely, therefore, to be because nitrate levels were below a threshold at which an association might be expected to be observed. In our analyses we found no evidence of an association between land use variables or pesticide use and type 1 diabetes, in keeping with the findings of an earlier study [48], although pesticide use in our study reflected agricultural use on farmland within each LAD, rather than household use, which may be a more direct and relevant metric.

### Strengths and limitations

Our large, national study benefits from wider exposure differentials than observed in the regional studies previously undertaken in the UK and elsewhere. Our small area approach might have reduced components of ecological bias owing to within-area heterogeneity, which might have affected previous assessments using countries as the unit of analysis. We applied an agnostic analytical approach (EnWAS), corrected for multiple testing, to assess a wide range of environmental variables, including many meteorological, demographic and pollution variables that have been found—often inconsistently—to associate with childhood type 1 diabetes. We employed multiple analytical approaches to assess the consistency and robustness of our findings.

Our HES-based incident dataset will likely include some readmissions (i.e. prevalent cases), because we were unable to exclude children who moved (and therefore changed postcode) before the introduction of the unique identifier HES-ID [14]. Some incident cases may be absent from our dataset, e.g. children treated outside the NHS setting. We have shown previously that HES-based data show good concordance with well-ascertained regional register data, especially from the year 2000 onwards, and for children aged 0–9 years [14], which influenced our choice of time period and age groups for this study. Type 1 diabetes is a heterogeneous disease [1, 49], and environmental triggers may differ in those with early onset vs those who present with diabetes at an older age. As noted above, we excluded children aged >9 years from our analysis but combined the 0–4 and 5–9 year age groups to ensure case counts per LAD were sufficient to deliver robust and interpretable results. Nonetheless, the LAD level sex-adjusted incidence rate for 0- to 4-year-olds vs 5- to 9-year-olds showed only a weak correlation (*r* = 0.35), as did age-adjusted LAD level incidence for boys vs girls (*r* = 0.24). Further analyses at a coarser geographic resolution might reveal different associations by age group and/or by sex, although with loss of spatial granularity in the exposure variables. The HES data capture details of diabetic individuals’ age and sex, admission date and diagnosis but do not provide any information on family history, lifestyle or diet, nor were area level data available for these variables. As such, analysis of the impact of these potential confounders on disease risk could not be assessed.

With respect to the meteorological, demographic and pollution variables investigated, these were available at a range of scales, and we had to average each to LAD level. Some variables, specifically radon potential class and pesticide use, were derived from data not ideally suited to the purpose. For other variables (e.g. air pollutants and population density), the aggregation to LAD level was at the loss of potentially important spatial granularity, which might have attenuated possible associations. While we tried to select environmental variables at or shortly before baseline (i.e. ~2000), this was not possible for all variables, including metals in soil and meteorological data, which were based on data collected over the study period and preceding decades, and the data on tobacco expenditure and ANGSt criteria, which were based on information which post-dates the end of the HES-based incident dataset. While this temporal mismatch between the environmental and health data might have introduced bias, the persistence in relative ranking at the LAD level of many of the studied environmental and demographic variables [50] suggests that any such bias is unlikely to have materially altered our findings.

As this is an area level study, ecological bias may be present, such that the ecological associations we observed may not reflect associations between these environmental variables and type 1 diabetes at the individual level. As many of the variables assessed were intercorrelated it was not possible to develop a full, mutually adjusted model; as such, confounding by measured and unmeasured variables (including diet, family history, etc.) may remain. We corrected for multiple testing and focused on those associations which were consistent across the EnWAS and disease-mapping analyses; nonetheless, there remains a risk of false-positive results and potentially important confounders which we could not assess. Replication in an independent cohort and/or in analyses undertaken at the individual level would be useful to validate and confirm these findings.

## Conclusions

Many demographic and environmental variables have been proposed as risk factors for childhood type 1 diabetes to explain disease onset and/or progression. Despite the many ecological and individual level epidemiological studies undertaken over the past 40 years, few variables have been consistently found to associate with type 1 diabetes. Our ecological EnWAS and disease mapping suggests a strong spatial structure in disease risk across England, with several environmental (PM_10_, nitrogen dioxide, nitrogen oxides, carbon monoxide, lead in soil, radon, outdoor light at night) and demographic (overcrowding, population density, ethnicity) variables associated with risk of type 1 diabetes. The geographic distribution of type 1 diabetes risk highlights rural and/or coastal areas as having higher risk, potentially supporting the hygiene hypothesis and/or indicating a protective effect of features associated with the urban environment and/or spatial variation in genetic susceptibility. Further investigation, at the individual level, may help identify modifiable environmental triggers of type 1 diabetes.

## Electronic supplementary material

ESM (PDF 222 kb)

## Data Availability

The majority of the demographic and environmental factors included in the study were derived from Crown copyright data and are available via the links provided in the text and references. HES data are available via NHS Digital on application with appropriate ethics and governance permissions; we do not hold data provider, ethics or governance permissions to share these health data with third parties.

## Abbreviations

ANGSt: Accessible natural greenspace standard
CrI: Credible interval
Defra: Department for Environment, Food and Rural Affairs
EnWAS: Environment-wide association study
HES: Hospital episode statistics
IMD: Index of Multiple Deprivation
LAD: Local authority district
NHS: National Health Service
PM_10_: Particulate matter with aerodynamic diameter <10 μm
PM_2.5_: Particulate matter with aerodynamic diameter <2.5 μm
