# Supplementary material for: Childhood type 1 diabetes: an environment-wide association study across England
Source: Diabetologia. 2020 Jan 24;63(5):964–76. doi: 10.1007/s00125-020-05087-7 (PMC7145790; doi:10.1007/s00125-020-05087-7)
Supplement: Supplementary file 1 — (PDF 222 kb) [file 125_2020_5087_MOESM1_ESM.pdf]

Childhood Type 1 Diabetes; an Environment Wide Association Study across England – Electronic Supplementary material

Annalisa Sheehan[1,2], Anna Freni Sterrantino[1,3], Daniela Fecht[1,3], Paul Elliott[1,3,4], Susan Hodgson[1,3]

1 MRC Centre for Environment and Health, Department of Epidemiology and Biostatistics, Imperial College London, London, UK

2 School of Population Health & Environmental Sciences, Faculty of Life Sciences & Medicine, King's College London, UK

3 UK Small Area Health Statistics Unit, School of Public Health, Imperial College London, London, UK

4 Imperial College NIHR Biomedical Research Centre, Imperial College London, London, UK

ESM Table 1: Summary of the environmental variables assessed in the ecological Environment Wide Association Study

Variable	Data source	Time period	Resolution	LAD-measure	25/50/75 percentile	Units
**Meteorological conditions**						
Sunshine duration (minimum)	British Atmospheric Data Centre/SAHSU	1980-2005	1×1km	Population weighted	3.45/3.74/ 3.90	Hours
Sunshine duration (maximum)	British Atmospheric Data Centre/SAHSU	1980-2005	1×1km	Population weighted	3.74/3.89/4.07	Hours
Minimum temperature	Met Office UKCP09	1981-2010	5km grid	Population weighted	0.51/0.97/1.43	^{o}C
Maximum temperature	Met Office UKCP09	1981-2010	5km grid	Population weighted	20.12/21.05/21.97	^{o}C
Ultraviolet Radiation	Cherrie et al. 2015[1]	July 2002-June 2003	postcode district	Area weighted	1857/2008/2102	J/m^2/day
**Built Environment**						
Green space	CEH Land Cover Map 2000	2000	25m×25m	Percent of total area	16.68/21.68/29.42	%
Blue space	CEH Land Cover Map 2000	2000	25m×25m	Percent of total area	0.29/0.72/2.55	%
Built environment	CEH Land Cover Map 2000	2000	25m×25m	Percent of total area	7.22/15.98/39.34	%
Intense agriculture	CEH Land Cover Map 2000	2000	25m×25m	Percent of total area	23.01/48.72/65.81	%
ANGSt criteria 1 - at least 2 hectares greenspace, no more than 300 metres (5 minutes walk) from home	OS Greenspace data	2017	-	Percent of population	31.70/39.98/49.71	%

[1] Cherrie MP, Wheeler BW, White PM, Sarran CE and Osborne NJ (2015). Coastal climate is associated with elevated solar irradiance and higher 25(OH)D level. Environment International 77:76-84

ANGSt criteria 2 - at least one accessible 20 hectare site within two kilometres of home	OS Greenspace data	2017	-	Percent of population	46.39/68.01/87.21	%
ANGSt criteria 3 - one accessible 100 hectare site within five kilometres of home	OS Greenspace data	2017	-	Percent of population	3.66/29.49/73.35	%
ANGSt criteria 4 - one accessible 500 hectare site within ten kilometres of home	OS Greenspace data	2017	-	Percent of population	0.00/0.00/0.00	%
Rural status	Defra's Rural Urban Classification	2001	-	Percent of population	1.50/28.88/66.70	%
Urban status	Defra's Rural Urban Classification	2001	-	Percent of population	33.29/71.12/98.50	%
**Environmental Pollutants**						
Aggregate radon potential class	PHE/BGS Radon Potential Dataset	2007	1km×1km	Population weighted	1/1/2	Radon Potential Class
$PM_{2.5}$	Defra	2002	1km×1km	Population weighted	9.00/9.63/10.33	$\mu g\ m^{-3}$
PM_{10}	Defra	2001	1km×1km	Population weighted	14.43/15.39/16.75	$\mu g\ m^{-3}$
NO_2	Defra	2001	1km×1km	Population weighted	19.44/26.12/31.79	$\mu g\ m^{-3}$
NOx	Defra	2001	1km×1km	Population weighted	28.71/44.12/58.66	$\mu g\ m^{-3}$
CO	Defra	2002	1km×km	Population weighted	0.20/0.24/0.28	$mg\ m^{-3}$
SO_2	Defra	2002	1km×km	Population weighted	2.44/3.40/4.88	$\mu g\ m^{-3}$
Ozone	Defra	2003	1km×1km	Population weighted	12.27/15.66/19.79	# days daily max 8-hr >120 $\mu g\ m^{-3}$
Benzene	Defra	2003	1km×1km	Population weighted	0.00/0.00/0.00	$\mu g\ m^{-3}$

PM_{10}	EU-wide LUR (Vienneau et al. 2013[2])	2007	100m resolution	Population weighted	20.65/21.83/23.24	µg m^{-3}
NO_2	EU-wide LUR (Vienneau et al. 2013[2])	2007	100m resolution	Population weighted	20.71/24.83/29.95	µg m^{-3}
Lead (Pb) in soil	BGS	1978-2014	Various (500m to 5km)	Population weighted	43.92/66.79/104	mg/kg
Cadmium (Cd) in soil	BGS	1978-2014	Various (500m to 5km)	Population weighted	0.21/0.32/0.49	mg/kg
Arsenic (As) in soil	BGS	1978-2014	Various (500m to 5km)	Population weighted	10.65/13.85/17.94	mg/kg
Nitrates in drinking water	14 Water companies[3]	2000-2010	Various	Population weighted	5.36/19.03/26.81	mg/l
Fungicides	INTRARESE project/Defra/FERA	June 2000	Ward 1998	Population weighted	0.60/46.05/439	Kg^2
Herbicides and desiccants	INTRARESE project/Defra/FERA	June 2000	Ward 1998	Population weighted	3.17/72.55/485	Kg^2
Growth regulators	INTRARESE project/Defra/FERA	June 2000	Ward 1998	Population weighted	0.00/36.72/263	Kg^2
Insecticides/nemacides/acaracides	INTRARESE project/Defra/FERA	June 2000	Ward 1998	Population weighted	0.27/5.70/34.37	Kg^2
Molluscicides and repellents	INTRARESE project/Defra/FERA	June 2000	Ward 1998	Population weighted	0.00/3.27/32.02	Kg^2
Other pesticides	INTRARESE	June	Ward 1998	Population	0.10/61.09/329	Kg^2

[2] Vienneau D, de Hoogh K, Bechle MJ, Beelen R, van Donkelaar A, Martin RV, Millet DB, Hoek G and Marshall JD (2013). Western European land use regression incorporating satellite- and ground-based measurements of NO2 and PM10. Environ Sci Technol 47(23): 13555-13564

[3] Anglian water, Bristol Water, South Staffs Water, Northumbrian Water, Portsmouth Water, Severn Trent Water, SES Water, Southern Water, South East Water, South West Water, Thames Water, United Utilities, Dŵr Cymru Welsh Water and Wessex Water

	project/Defra/FERA	2000		weighted		
Total pesticides	INTRARESE project/Defra/FERA	June 2000	Ward 1998	Population weighted	8.42/265/1622	Kg^2
Light at Night	MANTLE project (Briggs et al. 2007[4])	2002	200m raster grid	Population weighted	3.98/9.51/19.26	Deciles of arbitrary units
**Demographic**						
Population density 2000	Office for National Statistics	2000	LADUA 2001	1000 persons per km^2	0.17/0.50/1.84	persons per km^2
Population density 2001	Office for National Statistics	2001	LADUA 2001	1000 persons per km^2	0.18/0.50/1.84	persons per km^2
Overcrowding	Census 2001	2001	-	Percent of population	0.82/1.07/1.48	% ≥1 persons/room/ household
Index of Multiple Deprivation (IMD)	Communities and Local Government	2004	Super Output Area (SOA) 2001	Population weighted	12.20/16.89/23.82	IMD Score
IMD living environment domain	Communities and Local Government	2004	SOA 2001	Population weighted	11.19/15.87/23.96	IMD Score
IMD barriers to housing and services domain	Communities and Local Government	2004	SOA 2001	Population weighted	18.14/22.97/27.32	IMD Score
IMD education, skills and training deprivation domain	Communities and Local Government	2004	SOA 2001	Population weighted	12.59/17.82/24.73	IMD Score
IMD employment deprivation domain	Communities and Local Government	2004	SOA 2001	Population weighted	0.06/0.08/0.12	IMD Score
IMD income deprivation domain	Communities and Local Government	2004	SOA 2001	Population weighted	0.08/0.11/0.15	IMD Score
IMD crime domain	Communities and Local Government	2004	SOA 2001	Population weighted	-0.64/-0.29/0.16	IMD Score
IMD health deprivation and disability domain	Communities and Local Government	2004	SOA 2001	Population weighted	-0.68/-0.22/0.28	IMD Score

[4] Briggs DJ, Gulliver J, Fecht D and Vienneau DM (2007). Dasymetric modelling of small-area population distribution using land cover and light emissions data. Remote Sensing of Environment 108(4): 451-466

Tobacco expenditure	CACI	2014	COA 2011	Expenditure per adult (age ≥16 years)	6.25/6.96/7.71	£/week
White ethnicity	SAHSU	2001	Census Output Area (COA) 2001	Percent of population	93.94/97.66/98.62	%
Black ethnicity	SAHSU	2001	COA 2001	Percent of population	0.15/0.27/0.88	%
Asian ethnicity	SAHSU	2001	COA 2001	Percent of population	0.29/0.67/2.78	%

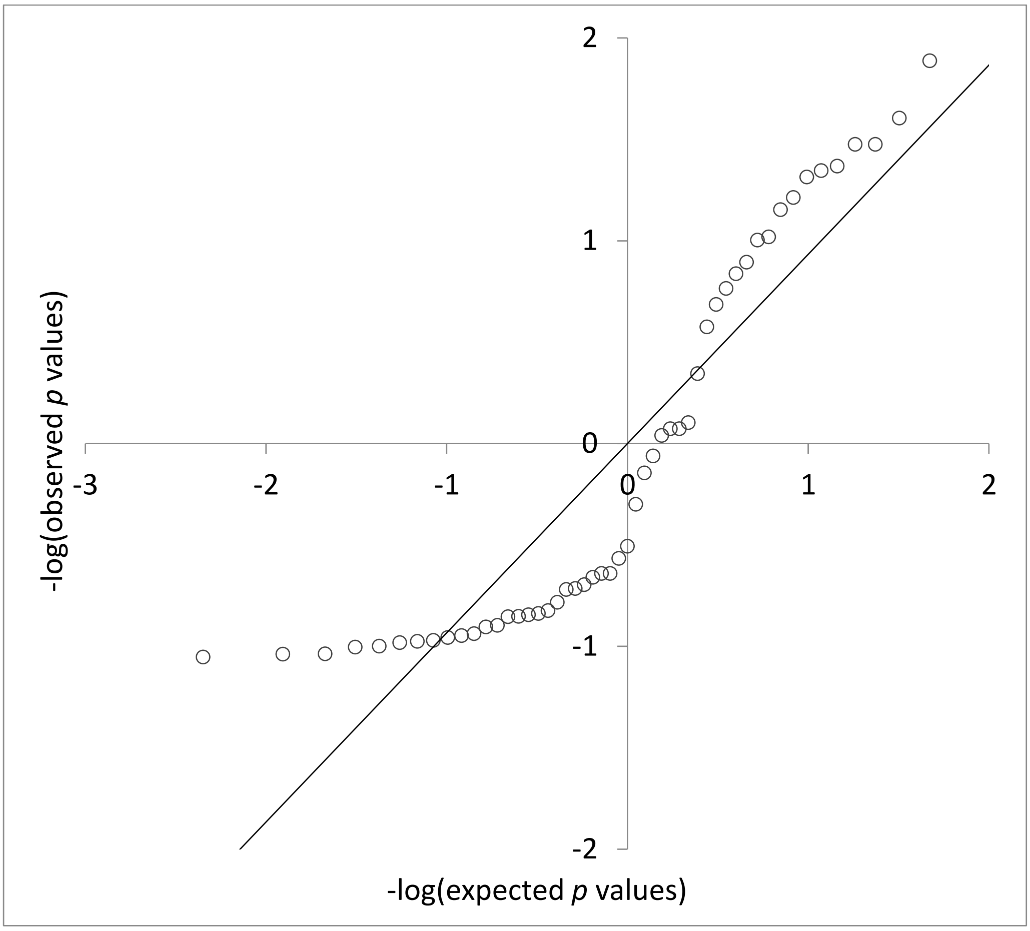

ESM Figure 1: Quantile-quantile (Q-Q) plot of the observed log *p* values (y axis) versus the expected log *p* values (y-axis) from the EnWAS. The diagonal (solid line) shows expectation under the null.
